# Development and external validation of the Psychosis Metabolic Risk Calculator (PsyMetRiC): a cardiometabolic risk prediction algorithm for young people with psychosis

**DOI:** 10.1016/S2215-0366(21)00114-0

**Published:** 2021-07

**Authors:** Benjamin I Perry, Emanuele F Osimo, Rachel Upthegrove, Pavan K Mallikarjun, Jessica Yorke, Jan Stochl, Jesus Perez, Stan Zammit, Oliver Howes, Peter B Jones, Golam M Khandaker

**Affiliations:** a Department of Psychiatry, University of Cambridge, Cambridge, UK; b Cambridgeshire and Peterborough NHS Foundation Trust, Cambridge, UK; c MRC London Institute of Medical Sciences, Institute of Clinical Sciences, Imperial College, London, UK; d Institute for Mental Health, University of Birmingham, Birmingham, UK; e Birmingham Women's and Children's NHS Trust Early Intervention Service, Birmingham, UK; f Department of Kinanthropology, Charles University, Prague, Czech Republic; g Centre for Academic Mental Health, Population Health Sciences, Bristol Medical School, University of Bristol, Bristol, UK; h MRC Integrative Epidemiology Unit, Population Health Sciences, Bristol Medical School, University of Bristol, Bristol, UK; i MRC Centre for Neuropsychiatric Genetics and Genomics, Cardiff University, Cardiff, UK; j Institute of Psychiatry, Psychology and Neuroscience, King's College London, London, UK

## Abstract

**Background:**

Young people with psychosis are at high risk of developing cardiometabolic disorders; however, there is no suitable cardiometabolic risk prediction algorithm for this group. We aimed to develop and externally validate a cardiometabolic risk prediction algorithm for young people with psychosis.

**Methods:**

We developed the Psychosis Metabolic Risk Calculator (PsyMetRiC) to predict up to 6-year risk of incident metabolic syndrome in young people (aged 16–35 years) with psychosis from commonly recorded information at baseline. We developed two PsyMetRiC versions using the forced entry method: a full model (including age, sex, ethnicity, body-mass index, smoking status, prescription of a metabolically active antipsychotic medication, HDL concentration, and triglyceride concentration) and a partial model excluding biochemical results. PsyMetRiC was developed using data from two UK psychosis early intervention services (Jan 1, 2013, to Nov 4, 2020) and externally validated in another UK early intervention service (Jan 1, 2012, to June 3, 2020). A sensitivity analysis was done in UK birth cohort participants (aged 18 years) who were at risk of developing psychosis. Algorithm performance was assessed primarily via discrimination (C statistic) and calibration (calibration plots). We did a decision curve analysis and produced an online data-visualisation app.

**Findings:**

651 patients were included in the development samples, 510 in the validation sample, and 505 in the sensitivity analysis sample. PsyMetRiC performed well at internal (full model: C 0·80, 95% CI 0·74–0·86; partial model: 0·79, 0·73–0·84) and external validation (full model: 0·75, 0·69–0·80; and partial model: 0·74, 0·67–0·79). Calibration of the full model was good, but there was evidence of slight miscalibration of the partial model. At a cutoff score of 0·18, in the full model PsyMetRiC improved net benefit by 7·95% (sensitivity 75%, 95% CI 66–82; specificity 74%, 71–78), equivalent to detecting an additional 47% of metabolic syndrome cases.

**Interpretation:**

We have developed an age-appropriate algorithm to predict the risk of incident metabolic syndrome, a precursor of cardiometabolic morbidity and mortality, in young people with psychosis. PsyMetRiC has the potential to become a valuable resource for early intervention service clinicians and could enable personalised, informed health-care decisions regarding choice of antipsychotic medication and lifestyle interventions.

**Funding:**

National Institute for Health Research and Wellcome Trust.

## Introduction

People with psychotic disorders such as schizophrenia have a life expectancy shortened by 10–15 years compared with the general population,^1^ predominantly owing to a higher prevalence of physical conditions such as type 2 diabetes, obesity, and cardiovascular disease (CVD).^2^ These comorbidities lead to a reduced quality of life and substantial health economic burden^3^ and usually develop early in the course of the psychotic disorder. For example, insulin resistance and dyslipidaemia are detectable from the onset of psychosis in adults in the second or third decades of life,^4^, ^5^ probably due to a combination of genetic, lifestyle, and other environmental influences.^6^ Since some treatments for psychosis can exacerbate cardiometabolic risk (eg, certain antipsychotic medications), identification of young adults at the highest risk of adverse cardiometabolic outcomes as soon as possible after diagnosis of a psychotic disorder is crucial, so that interventions can be tailored to reduce the risk of longer-term cardiovascular morbidity and mortality.

Prognostic risk prediction algorithms are a valuable means to encourage personalised, informed health-care decisions. In the general population, cardiometabolic risk prediction algorithms such as QRISK3^7^ are commonly used to predict CVD risk from baseline demographic, lifestyle, and clinical information, to identify higher-risk individuals for tailored interventions. A recent systematic review^8^ explored the suitability of existing cardiometabolic risk prediction algorithms for young people with psychosis. However, all algorithms were developed in samples of adults with a mean age across included studies of 50·5 years, and no studies included participants younger than 35 years. Most included studies did not include relevant predictors such as antipsychotic medication, so the authors of the review concluded that none are likely to be suitable for young people with psychosis.^8^ Furthermore, an accompanying exploratory analysis found that existing algorithms significantly underpredict cardiometabolic risk in young people with or at risk of developing psychosis.^8^

**Research in context**

**Evidence before this study**

Cardiometabolic risk prediction algorithms are commonly used in the general population as tools to encourage informed, personalised treatment decisions with the aim of primary prevention of longer-term cardiometabolic outcomes. In a recent systematic review of cardiometabolic risk prediction algorithms developed either for general or psychiatric populations, we searched Embase (1947 to Dec 1, 2019), Ovid MEDLINE (1946 to Dec 1, 2019), PsychINFO (1806 to Dec 1, 2019), Web of Science (from inception to Dec 1, 2019), and the first 20 pages of Google Scholar (to Dec 1, 2019). Search terms related to cardiometabolic (metabolism, metabolic, diabetes mellitus, cardiovascular disease, obesity, cardiometabolic); risk prediction (risk assessment, risk, outcome assessment, prediction, prognosis); and algorithm (calculator, computers, algorithms, software, tool) were included. Over 100 studies were included in the review. Yet, few were validated externally, only one was developed in a sample of people with mental illness, none were done in young populations, most were rated as being at high risk of bias, and most did not include relevant predictors such as antipsychotic medication. Additionally, existing algorithms substantially underpredict cardiometabolic risk in young people with or at risk of developing psychosis. Therefore, existing algorithms are unlikely to be suitable for young people with psychosis.

**Added value of this study**

We have developed and externally validated, to our knowledge, the first clinically useful and age-appropriate cardiometabolic risk prediction algorithm tailored for young people with psychosis—the Psychosis Metabolic Risk Calculator (PsyMetRiC)—using patient data from three geographically distinct UK National Health Service psychosis early intervention services. PsyMetRiC can reliably predict the risk of incident metabolic syndrome in young people with psychosis and young people who are at risk of developing psychosis.

**Implications of all the available evidence**

Whereas established risk prediction algorithms are suitable for use in older general population samples, with PsyMetRiC we are able to extend cardiometabolic risk prediction to young people with psychosis, a group who are at significantly higher cardiometabolic risk than the general population. Our findings can pave the way for a future clinical tool to encourage personalised treatment decisions with the aim of improving the long-term physical health of young people with psychosis.

Therefore, following TRIPOD reporting guidelines^9^ (appendix p 19), we developed and externally validated the Psychosis Metabolic Risk Calculator (PsyMetRiC) to predict up to 6-year risk of metabolic syndrome, an age-appropriate precursor of CVD and early mortality, in young people with psychosis. We prioritised clinical usefulness and patient acceptability via input from a young person's advisory group, and by developing two PsyMetRiC versions: one with and one without biochemical results.

## Methods

### Data sources

We developed PsyMetRiC using pooled retrospective data from patients aged 16–35 years enrolled in the Birmingham psychosis early intervention service (EIS; sample frame n=391) or Cambridgeshire and Peterborough Assessing, Managing and Enhancing Outcomes (CAMEO) EIS (sample frame n=1113). Anonymised data from the Birmingham psychosis EIS were collected between Jan 1, 2014, and Dec 31, 2018, as part of the National Clinical Audit of Psychosis Quality Improvement programme, and were enhanced locally with medication data conforming to the Health Research Authority definition of service evaluation, which were confirmed by Birmingham Women's and Children's Hospital National Health Service (NHS) Foundation Trust. CAMEO data were identified by anonymously searching for EIS patients enrolled between Jan 1, 2013, and Nov 4, 2020, using the Clinical Records Anonymisation and Text Extraction (CRATE) tool^10^ (NHS National Research Ethics Service references 12/EE/0407 and 17/EE/0442). Predictors were assessed at the closest point (within 100 days) to EIS enrolment, and outcomes were assessed up to 6 years later. We excluded patients who had less than 1 year of follow-up, had the outcome at baseline, or had missing data on all predictor or outcome variables.

To externally validate PsyMetRiC, we used the Clinical Records Interactive Search (CRIS) resource to capture anonymised data from South London and Maudsley NHS Foundation Trust (SLaM) EIS (National Institute for Health Research [NIHR] Biomedical Research Centre [BRC] CRIS Oversight Committee reference 20-005). Our sample frame included 2985 EIS patients aged 16–35 years enrolled between Jan 1, 2012, and June 3, 2020. Patients were excluded and predictors and outcomes were assessed in the same manner as for the development set.

In a sensitivity analysis, we examined the performance of PsyMetRiC in young adults who had or were at risk of developing psychosis from the Avon Longitudinal Study of Parents and Children (ALSPAC) birth cohort (appendix p 1).^11^ Our sample frame included participants identified as having experienced definite psychotic symptoms at either 18 years or 24 years, assessed via the semi-structured Psychosis-Like Symptom Interview (appendix p 1). Predictors were assessed at age 18 years, and the outcome was assessed at age 24 years. We excluded participants as described for the development set.

The ALSPAC Ethics and Law Committee and local research ethics committees provided ethical approval. Informed consent was obtained from patients following the recommendations of the ALSPAC Ethics and Law Committee at the time the data were collected.

### Outcomes

We used the harmonised definition^12^ of the metabolic syndrome as a binary outcome, which was at least three from the following list: ethnicity-specific waist circumference of at least 94 cm in males and at least 80 cm in females for white people, at least 90 cm in males and at least 80 cm in females for other ethnic groups, or body-mass index (BMI) greater than 29·9 kg/m^2^; triglyceride concentrations at least 1·70 mmol/L; HDL concentration less than 1·03 mmol/L in males or less than 1·29 mmol/L in females; systolic blood pressure greater than 130 mm Hg; or fasting plasma glucose greater than 5·60 mmol/L.

### Predictor variables

Predictors were included based on a balance of clinical knowledge, past research, likely clinical usefulness, and patient acceptability after discussion of the work with the McPin Foundation Young Persons Advisory Group (YPAG), a group of volunteers aged younger than 24 years with personal experience of mental health difficulties (appendix p 10). The full model comprised age (continuous; years), ethnicity (categorical; white European or not recorded [reference], Black or African-Caribbean, Asian, or other), sex (female or male), BMI (continuous; kg/m^2^), current smoking status (binary; at least one cigarette on average daily), prescription of a metabolically active antipsychotic drug (binary; based on relative cardiometabolic risk; appendix p 11), HDL concentration (continuous; mmol/L), and triglyceride concentration (continuous; mmol/L). A partial model, without HDL and triglyceride concentrations, was developed to cover eventualities where biochemical results are not available (appendix pp 5–8).

### Statistical analysis

We developed PsyMetRiC using the forced entry method, after ruling out predictor multi-collinearity, to minimise risk of overfitting and as recommended for smaller datasets.^13^ We did a formal sample size calculation.^14^ Briefly, the sample size required was estimated from the estimated outcome prevalence, the a priori estimated *R*^2^ of the model, and the estimated required model shrinkage. For the full model, the minimum sample required was 494, and for the partial model it was 394 (appendix p 2).^14^ We did not consider non-linear terms or interactions to reduce risk of overfitting. We used multiple imputation using chained equations for missing data and we pooled estimates using Rubin's rules (appendix p 3). An initial internal validation step (500 bootstraps) was done, and coefficients were shrunk for optimism using the pooled corrected C slope as a shrinkage factor. After this step, predictive performance was assessed (see later).

The algorithms were applied to the external validation sample. The distribution of predicted outcome probabilities was inspected using histograms. Algorithm performance was primarily assessed with measures of discrimination (C statistic) and calibration (calibration plots; appendix p 4). We also recorded Nagelkerke-Cox-Snell-Maddala-Magee *R*^2^ index, the calibration intercept (ideally close to 0), C slope (ideally close to 1), and the Brier score, which is an overall measure of algorithm performance (ideally close to 0, with scores >0·25 generally indicating a poor model).

Decision curve analysis^15^ was used to assess the clinical usefulness of PsyMetRiC by estimating net benefit. Net benefit is a metric of true positives minus false positives, and is calculated as

sensitivity×prevalence-(1-specificity)×(1-prevalence)×w

where *w* is the outcome odds at a given risk threshold.^16^ The risk threshold is the amount of tolerable risk before an intervention is deemed necessary. Net benefit incorporates the consequences of the decisions made on the basis of an algorithm, and is therefore preferable to related measures such as sensitivity and specificity alone.^16^ We also reported the standardised net benefit (net benefit/outcome prevalence) and related metrics (sensitivity and specificity). In decision curve analysis, consideration only of the range of risk thresholds that may reasonably be considered in clinical practice is customary. Our upper bound of 0·35 represents a greater than one in three chance of developing metabolic syndrome should nothing change, and it is unlikely that risk thresholds greater than this should be tolerated. We drew a decision curve plot to visualise the net benefit of both PsyMetRiC versions over varying risk thresholds compared with intervening in all patients or intervening in no-one. Net harm (ie, more false positives than true positives exposed to an intervention at a selected risk threshold) is indicated when a proposed intervention is plotted at y<0. Classical decision theory proposes that at a chosen risk threshold, the choice with the greatest net benefit should be preferred.^16^

### Visual representation of PsyMetRiC

We have provided two simulated case histories applying PsyMetRiC algorithms. Additionally, we developed an online data-visualisation app using shiny for R, which allows an interactive exploration of the effect of modifiable and non-modifiable risk factors and their combinations on cardiometabolic risk in young people with psychosis according to their PsyMetRiC score.

### Role of the funding source

The funders of the study had no role in study design, data collection, data analysis, data interpretation, or writing of the report.

## Results

Data from 651 patients were included in the pooled development sample: 352 from the Birmingham EIS and 299 from CAMEO (table 1). After 500 bootstraps, the pooled corrected C slope was 0·90 for the full model and 0·93 for the partial model; these values were used as shrinkage factors. Final PsyMetRiC coefficients are presented in table 2. Histograms showing the distribution of predicted outcome probabilities are provided in the appendix (p 14).

**Table 1:** Demographics and clinical characteristics of patients in the algorithm development and internal and external validation sets

		**Development sample**				
		Birmingham EIS (n=352)	CAMEO EIS (n=299)	Pooled development sample (n=651)	**SLaM EIS external validation sample (n=510)**	**ALSPAC risk of psychosis sensitivity analysis sample (n=505)**
Age, years		23·76 (4·90)	25·42 (4·77)	24·52 (4·91)	24·45 (4·75)	17·81 (0·43)
Ethnicity						
	White European or not recorded	111 (32%)	250 (84%)	361 (55%)	154 (30%)	494 (98%)
	Black or African-Caribbean	94 (27%)	15 (5%)	109 (17%)	250 (49%)	<5 (<1%)*
	Asian or other	147 (42%)	34 (11%)	181 (28%)	106 (21%)	<5 (<1%)*
Sex						
	Male	232 (66%)	208 (70%)	440 (68%)	351 (69%)	184 (36%)
	Female	120 (34%)	91 (30%)	211 (32%)	159 (31%)	321 (64%)
HDL concentration, mmol/L		1·76 (0·35)	2·08 (0·49)	1·88 (0·57)	1·57 (0·37)	1·21 (0·31)
Triglycerides concentration, mmol/L		1·46 (1·18)	1·30 (0·89)	1·39 (1·06)	1·23 (0·71)	1·06 (0·77)
BMI, kg/m^2^		22·06 (5·13)	24·01 (5·73)	23·63 (5·43)	22·96 (6·94)	23·22 (3·55)
FPG, mmol/L		5·20 (1·02)	5·17 (1·45)	5·19 (1·28)	5·03 (1·10)	5·31 (0·49)
Systolic BP, mm Hg		121·18 (11·04)	119·88 (12·25)	120·65 (11·68)	119·96 (13·70)	115·10 (11·88)
Metabolically active antipsychotics†		239 (68%)	216 (72%)	455 (70%)	472 (93%)	58 (11%)
Current smoker		182 (52%)	133 (44%)	315 (48%)	469 (92%)‡	286 (57%)
Follow-up, years		2·44 (1·54)	1·43 (1·03)	1·86 (1·32)	2·73 (1·76)	5·18 (0·39)
Time of predictor assessment from EIS enrolment, days		23·55 (25·44)	21·93 (29·84)	16·71 (26·38)	3·05 (36·01)	§
Metabolic syndrome at baseline¶		31/383 (8%)	18/317 (6%)	49/700 (7%)	30/540 (6%)	22/527 (4%)
Metabolic syndrome at follow-up		74 (21%)	35 (12%)	109 (17%)	86 (17%)	76 (15%)

Data are mean (SD), number (%), or n/N (%). Some percentags do not add up to 100 because of rounding. ALSPAC=Avon Longitudinal Study of Parents and Children. BMI=body-mass index. BP=blood pressure. CAMEO=Cambridgeshire and Peterborough Assessing, Managing and Enhancing Outcomes. EIS=early intervention service. FPG=fasting plasma glucose. SLaM=South London and Maudsley NHS Foundation Trust.

* Reported as <5 owing to ALSPAC reporting guidelines.

† Listed in the appendix (p 11).

‡ Smoking status was derived using the CRIS-IE-Smoking application using natural language processing software to extract ever smoking status information from open-text fields (appendix p 6).

§ Health record and service use data are not available in ALSPAC.

¶ N numbers are the sample size before excluding cases with metabolic syndrome at baseline.

**Table 2:** Final coefficients for the Psychosis Metabolic Risk Calculator after shrinkage for optimism

	**Full model**	**Partial model**
Intercept	−6·439813	−6·973829
Age, years	0·006233226	0·00633115
Black or African-Caribbean ethnicity	0·004258861	0·07548129
Asian or other ethnicity	0·211217746	0·29285950
Male sex	0·222300765	0·31460036
Body-mass index, kg/m^2^	0·141186241	0·16912161
Current smoker	0·153691193	0·24751854
Prescribed a metabolically active antipsychotic	0·497552758	0·60013558
HDL, mmol/L	−0·399013329	*
Triglycerides, mmol/L	0·343528440	*

* Variable not included in model.

At internal validation, the pooled performance statistics for the full model were C 0·80 (95% CI 0·74 to 0·86); *R*^2^ 0·25 (95% CI 0·22 to 0·28); Brier score 0·07 (95% CI 0·05 to 0·09); and intercept −0·05 (95% CI −0·08 to −0·02). For the partial model, these statistics were C 0·79 (95% CI 0·73 to 0·84); *R*^2^ 0·19 (95% CI 0·14 to 0·24); Brier score 0·10 (95% CI 0·07 to 0·13); and intercept −0·07 (95% CI −0·10 to −0·04). Calibration plots showed good agreement between observed and expected risk at most predicted probabilities, although in both PsyMetRiC versions there was evidence of slight overprediction of risk at higher predicted probabilities (appendix p 15).

**Figure 1:**
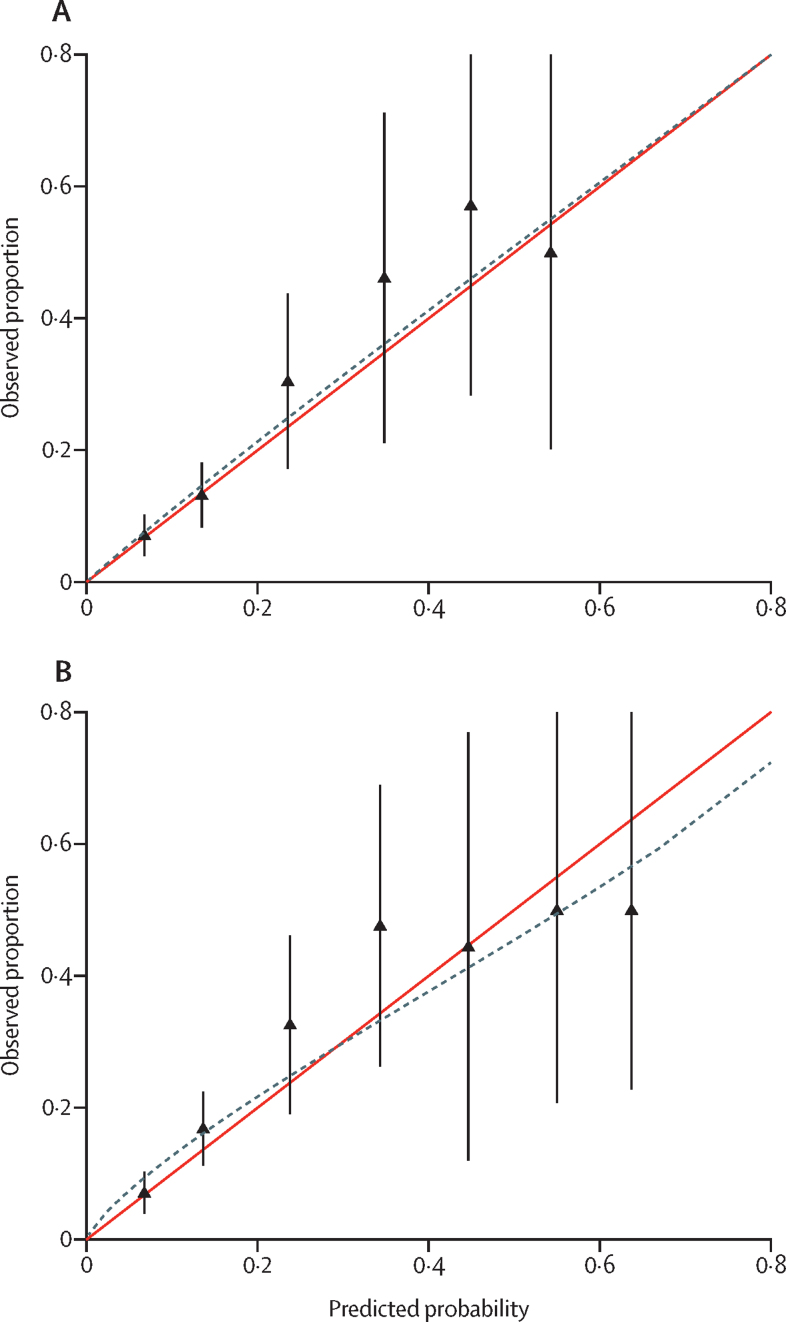
Calibration plots for external validation of PsyMetRiC algorithms in an early intervention service patient sample Calibration plots are shown for the PsyMetRiC full model (A) and partial model (B). Calibration plots illustrate agreement between observed risk (y axis) and predicted risk (x axis). Perfect agreement would trace the red line. Algorithm calibration is shown by the dashed line. Triangles denote grouped observations for participants at deciles of predicted risk, with 95% CIs indicated by the vertical black lines. Axes range between 0 and 0·8 since very few individuals received predicted probabilities greater than 0·8. PsyMetRiC=Psychosis Metabolic Risk Calculator.

Our sample frame in the SLaM EIS identified 2985 patients, 510 of whom were eligible for inclusion in the SLaM external validation set; the appendix (p 9) provides details of the missing sample analysis. After applying PsyMetRiC to the SLaM EIS patient sample, performance statistics for the full model were C 0·75 (95% CI 0·69 to 0·80); *R*^2^ 0·21 (95% CI 0·18 to 0·25); Brier score 0·07 (95% CI 0·04 to 0·10); and intercept −0·05 (95% CI −0·08 to −0·02). For the partial model, these statistics were C 0·74 (95% CI 0·67 to 0·79); *R*^2^ 0·17 (95% CI 0·14 to 0·20); Brier score 0·08 (95% CI 0·05 to 0·11); and intercept −0·07 (95% CI −0·11 to −0·03). Calibration plots showed good agreement between observed and expected risk in the full model, but in the partial model there was evidence of slight miscalibration (underprediction of risk at lower predicted probabilities, and overprediction of risk at higher predicted probabilities; figure 1). In both models, 95% CIs widened as predicted probabilities became more extreme owing to lower numbers of participants with more extreme predicted probabilities (appendix p 15).

The sample frame for the ALSPAC validation set comprised 505 patients. In the ALSPAC sample, performance statistics for the full model were C 0·73 (95% CI 0·66 to 0·79); *R*^2^ 0·20 (95% CI 0·17 to 0·23); Brier score 0·08 (95% CI 0·04 to 0·11); and intercept −0·03 (95% CI −0·07 to 0·01). For the partial model, these statistics were C 0·71 (95% CI 0·64 to 0·77); *R*^2^ 0·17 (95% CI 0·13 to 0·22); Brier score 0·09 (95% CI 0·05 to 0·13); and intercept −0·03 (95% CI −0·07 to 0·00). The appendix (p 17) shows histograms of predicted outcome probabilities. Calibration plots showed good agreement between observed and expected risk in the full model, albeit with some minor evidence of miscalibration (slight underprediction of risk at lower predicted probabilities, and overprediction of risk at higher predicted probabilities; appendix p 18). The same pattern of slight miscalibration was marginally more pronounced in the partial model.

**Figure 2:**
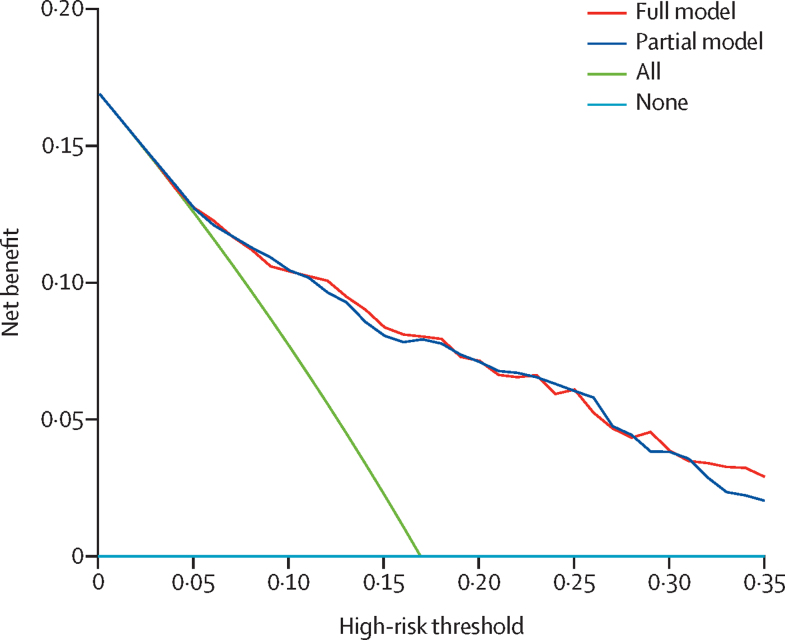
Decision curve analysis plot for PsyMetRiC full and partial models The plot reports net benefit (y axis) of PsyMetRiC full and partial models across a range of risk thresholds (x axis) compared with intervening in all patients or intervening in no patients. PsyMetRiC=Psychosis Metabolic Risk Calculator.

Decision curve analysis suggested that at predicted probability cutoffs greater than 0·05, both PsyMetRiC algorithms provided greater net benefit than the competing extremes of intervening in all patients or in none (figure 2). At most risk thresholds greater than 0·05, the full model provided slight improvement in net benefit compared with the partial model. The appendix (pp 12–13) provides numerical decision curve analysis results (net benefit, standardised net benefit, sensitivity, and specificity) across a range of reasonable risk thresholds. For example, if an intervention were considered necessary above a risk score of 0·18, the full model would provide a net benefit of 7·95% (95% CI 5·37–10·82), with a sensitivity of 75% (95% CI 66–82) and specificity of 74% (71–78), meaning that an additional 47% of metabolic syndrome cases could be prevented (standardised net benefit). At the same risk threshold, the partial model would provide a net benefit of 7·74% (95% CI 4·79–10·36), with a sensitivity of 75% (95% CI 65–81) and specificity of 74% (70–77), meaning that an additional 46% of metabolic syndrome cases could be prevented (standardised net benefit). For both models, these data equate to around an additional eight cases of metabolic syndrome that could be prevented per 100 individuals, without any increase in false positives.

**Figure 3:**
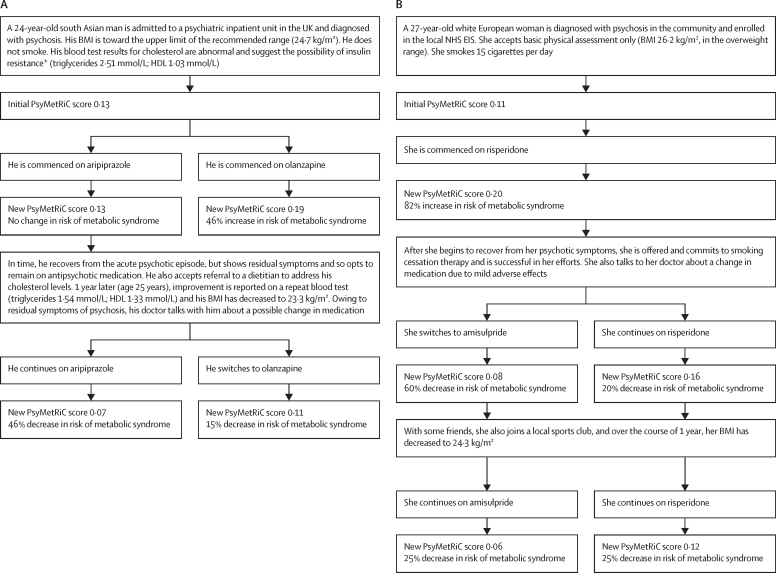
Simulated case scenarios to visualise the effect of modifiable and non-modifiable risk factors on cardiometabolic risk in young people with psychosis as calculated from PsyMetRiC full and partial models Case scenarios are shown for the PsyMetRiC full model (A) and partial model (B). PsyMetRiC scores are presented as predicted probabilities, which can be converted to percentage chance of incident metabolic syndrome by multiplying by 100. BMI=body-mass index. EIS=early intervention service. NHS=National Health Service. PsyMetRiC=Psychosis Metabolic Risk Calculator. *A raised triglyceride:HDL ratio is indicative of insulin resistance.^17^

Figure 3 shows decision trees outlining two simulated case scenarios to visualise the effect of modifiable and non-modifiable risk factors in young people with psychosis, as calculated from PsyMetRiC full and partial models. We have developed an online data visualisation app for both PsyMetRiC versions, which allows the user to interactively explore the effect of modifiable and non-modifiable risk factors and their combinations on cardiometabolic risk in young people with psychosis, based on PsyMetRiC scores.

## Discussion

We have developed and externally validated PsyMetRiC, which is to our knowledge the first cardiometabolic risk prediction algorithm tailored specifically for young people with psychosis. PsyMetRiC can predict up to 6-year risk of incident metabolic syndrome from commonly recorded clinical information, highlighting modifiable risk factors that could be addressed to reduce risk. Metabolic syndrome is a precursor to CVD and early mortality,^18^ and is a suitable outcome for younger populations, since it occurs more commonly in younger adults than do more distal cardiovascular endpoints such as CVD. The external validation of both PsyMetRiC versions was good, with C statistics greater than 0·70. Calibration of the full model was good, but there was evidence of slight miscalibration of the partial model. Therefore, the partial model in particular may benefit from recalibration in larger samples. Both PsyMetRiC versions displayed greater net benefit than alternative strategies across a range of feasible risk thresholds, although at most risk thresholds our results show that the full model should be used preferentially.

Our data visualisations help to illustrate three things: first, antipsychotic medication choice imparts a substantial influence on cardiometabolic risk; second, addressing lifestyle factors can effectively reduce cardiometabolic risk even in the presence of antipsychotic medication; and third, advancing age in young adults does not influence cardiometabolic risk substantially relative to other risk factors. Although PsyMetRiC will benefit from future validation in larger samples, it has the potential to become a valuable resource to promote better management of physical health in young people with psychosis—eg, by highlighting modifiable risk factors and encouraging clinicians to make more personalised, informed decisions, such as with the choice of antipsychotic medication or lifestyle interventions, or both.

Ethnicity, smoking, and BMI are among the most commonly included predictors in existing algorithms^8^ and are well known contributors to cardiometabolic risk,^19^ so we included them in PsyMetRiC. Sex is also frequently considered in existing algorithms,^8^ and we included it in PsyMetRiC. We found that male sex was a risk factor for incident metabolic syndrome, which aligns with meta-analytic reports that male sex is a risk factor for antipsychotic-induced metabolic dysfunction.^19^ Our available sample size was too small to be able to consider separate versions of PsyMetRiC for males and females. If larger samples become available in the future, sex-stratified versions could be considered, since existing algorithms developed for the general population commonly take this step.^8^

Age is frequently included in existing algorithms,^8^ and we included it in PsyMetRiC. However, existing cardiometabolic risk prediction algorithms, which were developed for older adults, weighted age to a greater extent than other predictors.^8^ This is probably because most cardiometabolic risk factors contribute cumulative risk over time;^20^ thus, age becomes increasingly important as one gets older. A recent exploratory analysis^8^ that examined the predictive performance of the existing general population cardiometabolic risk prediction algorithms, including QRISK3^7^ and PRIMROSE,^21^ in young people who were at risk of developing psychosis found that each significantly underpredicted risk in the younger population, possibly owing to the way existing algorithms have modelled age. For example, in PsyMetRiC, age is weighted to a much lesser extent than other predictors, and we achieved favourable calibration in younger populations. Although QRISK3^7^ and PRIMROSE^21^ are good examples of well designed algorithms from large samples, our results suggest that PsyMetRiC is more appropriate for young people with psychosis.

Blood-based predictors, such as HDL and triglyceride concentrations, feature relatively infrequently in cardiometabolic risk prediction algorithms.^8^ Meta-analytic evidence suggests abnormal triglyceride and HDL concentrations are detectable at first-episode psychosis,^22^ and a raised triglyceride:HDL ratio is a hallmark of insulin resistance,^23^ which is also associated with first-episode psychosis.^4^ Abnormal HDL and triglyceride concentrations are associated longitudinally with cardiometabolic outcomes.^24^ Guideline recommendations encourage blood-based monitoring both before and after antipsychotic exposure,^25^ and so such data should be available. We found that the inclusion of blood-based predictors improved all predictive performance metrics. However, blood-based monitoring might not always be possible, and we found that the partial model still provided reliable performance estimates, although it would benefit from recalibration.

Antipsychotic medication is an important contributor to cardiometabolic risk in young people with psychosis, yet has rarely been included in existing algorithms. Some recent algorithms have included antipsychotics as predictors, grouped according to the traditional distinctions of typical and atypical or first and second generation.^8^ However, the differential cardio-metabolic effects of antipsychotics do not abide by these distinctions. Therefore, we instead modelled antipsychotics based on previous research (appendix p 11).

PsyMetRiC cannot yet be recommended for clinical use and requires prospective validation in larger samples, health technology assessment, and regulatory approval. However, in the future, PsyMetRiC could become a useful resource for the improved management of physical health in young people with psychosis. For example, in the presence of a very low PsyMetRiC risk score, gentle encouragement to maintain good physical health might be sufficient. This might include dietary advice or promoting daily physical activity and smoking cessation, if necessary, or both. There is little harm, yet much to gain, in offering gentle encouragement to live a healthier life, and such conversations need to become part of psychiatric consultation.

Patients and clinicians might prefer to tolerate a slightly higher threshold of risk when the proposed intervention could be deemed more burdensome or might increase the risk of other adverse effects. Regarding interventions that might be deemed more burdensome, prescribed lifestyle interventions have shown promise in lowering cardiometabolic risk in young people with psychosis,^17^ but regular appointments may be difficult to maintain around work or other commitments. Regarding interventions that might increase the risk of other adverse effects, our results show that switching from metabolically active antipsychotics, or not prescribing them in the first place, is an effective means to reduce cardiometabolic risk. However, the risk of psychosis relapse or other adverse effects might reasonably be worrisome for patients and clinicians alike. Moreover, data from a meta-analysis^19^ suggest that metabolically active antipsychotics could be associated with greater psychosis treatment response. Therefore, antipsychotic selection must strike an intricate balance between caring for psychiatric and physical health. Finally, trials of treatments such as metformin and statins are scarce in young people with psychosis, but evidence suggests that such medications might benefit both cardiometabolic and psychiatric outcomes.^26^

We have developed, to our knowledge, the first cardiometabolic risk prediction algorithm for young people with psychosis, harnessing data from three geographically distinct patient samples and a population-based cohort. PsyMetRiC was developed in consultation with The McPin Foundation YPAG to ensure balance between clinical practicality and patient acceptability, and we received encouraging comments from the YPAG about PsyMetRiC (appendix p 10). We developed an online interactive app permitting a visualisation of the effect of different cardiometabolic risk factors in young people with psychosis. We have published our algorithm coefficients to encourage future validation and updating. We developed two versions of PsyMetRiC to maximise clinical utility and both validated well, suggesting that PsyMetRiC is likely to be suitable for use in patients aged 16–35 years from a UK EIS population, and, from the results of our sensitivity analysis, for use in young adults at risk of developing psychosis.

Limitations of the study include missing data. We excluded participants who had the outcome at baseline, as recommended;^27^ however, since predictors were assessed within a short timeframe after EIS enrolment, some metabolically sensitive individuals might have been excluded from our analysis. We also excluded participants with data missing on either all exposure or all outcome variables, which might also have introduced selection bias. The missing samples were more likely to be older and female, and less likely to be prescribed metabolically active antipsychotics. These factors might have affected some PsyMetRiC predictor coefficients. Nevertheless, we felt this exclusion step was more appropriate than imputing complete participant data. Multiple imputation can be biased when data are missing not at random, although we included auxiliary variables to reduce the fraction of missing information, limiting the effect of this bias. External validation of PsyMetRiC on larger samples is required since simulation studies have suggested a minimum of 100 outcome events for an accurate validation analysis.^28^ Larger prospectively collected samples in future might also allow for updating the algorithm with interactions, non-linear terms, sex stratification, and other potentially important predictors such as other metabolically active medications, physical activity, and diet. Prospectively collected data might also predict longer-term risk. The samples in our main analysis had outcomes measured up to 6 years; however, the mean follow-up time was shorter. Although our data-driven classification of metabolically active antipsychotics is an advance over existing algorithms, the metabolically active nature of different antipsychotics lies on a continuum rather than a dichotomy. Larger samples might permit the modelling of antipsychotics individually. Prescriber bias might have downwardly biased the coefficients for antipsychotics, since metabolically active medications might have been withheld from patients considered to be at higher cardiometabolic risk.

PsyMetRiC has the potential to become a valuable resource for health-care professionals working in EISs by aiding the informed choice of antipsychotic medication, prescription of cardioprotective drugs, and non-pharmacological interventions including lifestyle adjustments to prevent the future development of cardiometabolic comorbidities and consequent years of life lost.

For **ALSPAC grant details** see http://www.bristol.ac.uk/alspac/external/documents/grant-acknowledgements.pdf

## Data sharing

The data used in this study cannot be publicly deposited owing to patient and participant confidentiality reasons. CRIS and CRATE electronic health record data can be accessed after formal application to and ethical review by the Cambridgeshire and Peterborough NHS Foundation Trust and the South London and Maudsley NHS Foundation Trust, respectively. Access to ALSPAC data can be made following formal application to the ALSPAC executive committee. See http://www.bris.ac.uk/alspac/researchers/data-access/data-dictionary/ for a fully searchable data dictionary for ALSPAC.

## Declaration of interests

BIP reports a fellowship grant from the NIHR during the conduct of this study. RU reports personal fees from Sunovion, outside the submitted work. PKM reports personal fees from Recordati and Sunovion, outside the submitted work. OH reports grants and personal fees from Angelini, Autifony, Biogen, Boehringer Ingelheim, Eli Lilly, Heptares, Global Medical Education, Invicro, Janssen, Lundbeck, Mylan, Neurocrine, Otsuka, Sunovion, Rand, Recordati, and Roche, outside the submitted work. All other authors declare no competing interests.
