# Supplementary material for: Development and external validation of the Psychosis Metabolic Risk Calculator (PsyMetRiC): a cardiometabolic risk prediction algorithm for young people with psychosis
Source: Lancet Psychiatry. 2021 Jul;8(7):589–98. doi: 10.1016/S2215-0366(21)00114-0 (PMC8211566; doi:10.1016/S2215-0366(21)00114-0)
Supplement: Supplementary appendix [file mmc1.pdf]

THE LANCET
Psychiatry

Supplementary appendix

This appendix formed part of the original submission and has been peer reviewed. We post it as supplied by the authors.

Supplement to: Perry BI, Osimo EF, Upthegrove R, et al. Development and external validation of the Psychosis Metabolic Risk Calculator (PsyMetRiC): a cardiometabolic risk prediction algorithm for young people with psychosis. *Lancet Psychiatry* 2021; published online June 1. http://dx.doi.org/10.1016/S2215-0366(21)00114-0.

Development and External Validation of The Psychosis Metabolic Risk Calculator (PsyMetRiC): A Cardiometabolic Risk Prediction Algorithm for Young People with Psychosis

Perry B.I. et al

Appendix

Supplementary Methods

Sensitivity Analysis in The Avon Longitudinal Study of Parents and Children (ALSPAC) Birth Cohort

We examined the performance of PsyMetRiC in young adults who had or were at risk of developing psychosis, from the Avon Longitudinal Study of Parents and Children (ALSPAC) birth cohort[1-3]. ALSPAC initially recruited 14,541 pregnant women from southwest England in 1991/1992, and 13,988 children were alive at 1y. After age 7y, 913 additional participants were recruited. Our sample frame included 527 participants identified as having experienced definite psychotic symptoms at either 18/24y, assessed via the semi-structured Psychosis-Like Symptom Interview (Supplementary Methods). Predictors were assessed at 18y, and the outcome was assessed at 24y. We excluded participants as described above, resulting in a final sample of *n*=515 (Table 1). Data were managed using REDCap (University of Bristol[4,5]). ALSPAC Ethics and Law Committee and Local Research Ethics Committees provided ethical approval. Informed consent was obtained from participants following the recommendations of the ALSPAC Ethics and Law Committee at the time.

ALSPAC Sensitivity Analysis Sample – Psychosis-Like Symptom Interview

Psychotic Experiences

Psychotic Experiences were identified[6] through the face-to-face, semi-structured Psychosis-Like Symptom Interview (PLIKSi) conducted by trained psychology graduates and coded as per the definitions in the Schedules for Clinical Assessment in Neuropsychiatry, Version 2.0. The PLIKSi comprised of an introductory set of questions on unusual experiences, and then 12 ‘ core’ questions eliciting key symptoms covering the three main domains of positive psychotic symptoms: hallucinations (visual and auditory); delusions (delusions of being spied on, persecution, thoughts being read, reference, control, grandiose ability and other unspecified delusions); and symptoms of thought interference (thought broadcasting, insertion and withdrawal). After cross-questioning, interviewers rated PEs as not present, suspected, or definite. We included cases of definite PEs; the comparator group was suspected/no PEs.

We used data collected at ages 18 and 24 years. For interrater reliability, the interviewers recorded audio interviews at three time points, approximately 6 months apart, across the clinic duration (75 interviews in total). At age 18, the average kappa value of PEs was 0.83, with no evidence of differences across time. Test-retest reliability was assessed using 162 individuals reinterviewed after approximately 47 days (kappa=0.76, SE=0.078), 46 of whom were reinterviewed by the same interviewer (kappa=0.86, SE=0.136)[7]. At age 24, the PLIKSi had good interrater reliability (Intraclass correlation: 0.81; 95% CI, 0.68-0.89) and test-retest reliability (0.90; 95% CI 0.83-0.95)[6].

Sample Size Calculation for Development of a Prediction Algorithm

Riley and colleagues[8] proposed a set of criteria that sample size should meet for development of a prediction algorithm (binary outcome), in order to minimise the risk of overfitting and to ensure precise estimation of key parameters in the prediction algorithm. The sample size calculation requires the user-specified anticipated R^2 of the algorithm, and the average outcome value and standard deviation of outcome values in the population of interest. The three criteria are:

a) small overfitting defined by an expected shrinkage of predictor effects by 10% or less.

b) small absolute difference of 0.05 in the algorithm's apparent and adjusted Nagelkerke's R-squared value.

c) precise estimation (within +/- 0.05) of the average outcome risk in the population.

Three calculations of sample size are made based upon these criteria. The final recommended sample size is taken as the largest of the three individual calculations. See Riley and colleagues[8] for more information.

Sample Size Calculation for PsyMetRiC

The above criteria have been developed into a statistical package, *pmsampsize*[9] for R[10], which we used for sample size calculation. The user-specified arguments were:

1) Outcome prevalence = 20% based on meta-analytic prevalence estimates of unmedicated psychosis patients[11].
2) R^2 = 0.15, selected as a conservative estimate since there is no equivalent risk prediction algorithm developed in the same population with which to base the calculation. We did not consider using the one previous cardiovascular risk prediction algorithm developed for people with serious mental illness[12] to derive our calculation since the sample size was very large, and the algorithm was developed in a much older population, and with a different outcome. Should we have used their estimate (C=0.80, converted using Table 2 from Riley and colleagues[8] to R^2=0.47), our sample size requirement would have been significantly smaller.
3) Shrinkage = 0.9 (as recommended[9]).

Results of Sample Size Calculations for PsyMetRiC using pmsampsize[9] based on criteria proposed by Riley et al[8]

Criteria	Sample Size	Shrinkage	Parameters	R^2	Events Per Predictor
**Full-Model**					
Criteria 1	494	0.90	9	0.15	10.98
Criteria 2	259	0.83	9	0.15	5.76
Criteria 3	246	0.90	9	0.15	5.47
Final	494	0.90	9	0.15	10.98
**Partial-Model**					
Criteria 1	384	0.90	7	0.15	10.97
Criteria 2	201	0.83	7	0.15	5.74
Criteria 3	246	0.90	7	0.15	7.03
Final	384	0.90	7	0.15	10.97

Missing Data

We used multiple imputation using chained equations[13] (MICE) for missing data in all samples for predictors which were <40% missing[14] and had suitable auxiliary variables available for use as 'indicators of missingness' to reduce the impact of 'missing not at random' bias[15]. We imputed 100 datasets. Auxiliary variables were selected based upon minimizing the fraction of missing information[16]. Box-and-Whisker and Density plots were used to check similarities of observed and imputed data. Estimates were pooled using Rubin's rules.

Proportion of Missing Data per Variable for Model Development and External Validation

Variable	Model Development Sample	External Validation Sample
Sex	0	0
Ethnicity	0	0
Smoking Status	0	0
Age	0	0
Antipsychotic Prescription	0	0
SBP – Baseline	0.11	0.09
SBP – Follow-up	0.38	0.09
BMI – Baseline	0.32	0.17
BMI – Follow-up	0.31	0.13
Triglycerides – Baseline	0.33	0.16
Triglycerides – Follow-up	0.37	0.20
HDL – Baseline	0.33	0.16
HDL – Follow-up	0.37	0.20

Algorithm Performance

The C-statistic is derived from the area under the curve (AUC), and estimates the probability that a randomly selected 'case' will have a higher predicted probability for incident metabolic syndrome than a randomly selected non-case. Scores of 1.0 indicate perfect discrimination; scores of 0.5 indicate that the algorithm is no better than chance; scores of >0.7 are generally considered acceptable[17]. Calibration plots estimate the accuracy of absolute-risk estimates (i.e. agreement between observed and predicted risk).

Rationale and Coding of Predictors Included in PsyMetRiC

Age

Age is frequently included in existing cardiometabolic risk prediction algorithms[18], and we also included it in PsyMetRiC as a continuous variable. Whilst some previous large-scale general population risk-prediction algorithms have considered age either as a non-linear term or as an interaction term with other predictors[18], we did not take this step in order to limit potential model complexity and thus reduce the risk of model-overfit given our sample size. Considering age as an interaction term with other predictors would have added the requirement for a variable selection technique such as backward selection, or more automatic penalized methods such as lasso regression with nested cross-validation. Given our limited sample size, we chose not to proceed with such methods since they increase the risk of model overfit in smaller samples compared with our method of forced-entry[19-21], and thus may have hampered external validation performance and thus generalizability of PsyMetRiC[22].

Ethnicity

Ethnicity is one of the most frequently included predictors in existing cardiometabolic risk prediction algorithms[18]. Non-White ethnicity is an important risk factor for metabolic syndrome[23], and is a predictor of antipsychotic-induced metabolic dysfunction[24]. In a previous UK population-based study, South Asian ethnicity has shown the highest risk for metabolic syndrome, followed by Black/African-Caribbean ethnicity, followed by White European ethnicity[25]. In other studies, East Asian ethnicity has conferred significant risk of metabolic syndrome[26]. In our development and validation samples, ethnicity was recorded inconsistently, with the majority of included records classified in relatively simple terms, for example “White”, or “Asian”. However, these simplified classifications do not recognise the heterogeneity which may exist within these groupings, therefore potentially incorrectly inferring that the populations are homogeneous[27]. Nevertheless, to strike an appropriate balance between our available sample size, the case-mix of our development and validation samples, and with a consideration to maximise coding harmonisation between datasets, we proceeded with a categorical nominal variable with as much granularity as the data permitted, and so our variable consisting of White European/not stated (reference category), Black/African-Caribbean ethnicity, and Asian/Other ethnicity.

Sex

Sex is frequently considered in cardiometabolic risk prediction algorithms, either as a predictor or a stratification variable[18], and so we included it in PsyMetRiC. There are well-known sex differences in the epidemiology, aetiology, biology and clinical expression of metabolic syndrome[28]. For example, before the menopause, increased adiposity is more commonly precipitated in females than males[29], whereas hypertension and disrupted biochemical indices are more common in males[30], possibly due to a metabolically-active effect of oestrogen[31]. Longer-term cardiovascular outcomes such as CVD affect both sexes but also show differences in presentation and clinical course[32]. Recent meta-analytic reports have suggested that male sex is an important risk factor for antipsychotic-induced biochemical disruption[24]. Considering our available sample size, we did not consider separate algorithms for males and females, and so chose to model sex as a binary variable.

Body Mass Index

Body Mass Index (BMI) is frequently included in cardiometabolic risk prediction algorithms[18], and overweight/obesity is a reliable predictor of adverse cardiometabolic and cardiovascular outcomes[33]. Weight gain is also a common side-effect of certain antipsychotic medications[34] and can precipitate relatively quickly after initiation[35]. Whilst BMI may be less accurate at classifying adiposity compared with laboratory or research-based measures such as dual-energy x-ray absorptiometry or bio-impedance analysis[36], it is commonly recorded in clinical practice and correlates well with other measures of obesity[37]. Therefore, we included BMI as a continuous variable. We did not consider interactions of BMI with other predictors (including but not limited to, for example, antipsychotic medication) in order to limit potential model complexity and thus reduce the risk of model-overfit in our relatively small sample.

Smoking

Smoking is frequently included in cardiometabolic risk prediction algorithms[18], and is strongly associated with adverse cardiometabolic and cardiovascular outcomes[38]. The impact of smoking on cardiometabolic and cardiovascular risk is dose-dependent, yet, in previous large-scale general population algorithms developed for older adult populations, smoking has usually been classified as a categorical variable including ‘current smoker’, ‘ex-smoker’ and ‘never-smoked’[18]. The lack of consideration of dosage in previous algorithms (i.e., the number of cigarettes smoked per day and for how long) is likely due to the highly variable reporting of smoking history in electronic health record datasets[39]. However, whilst a prolonged smoking history increases cardiometabolic and cardiovascular risk compared with ‘never smoked’[40], particularly in older adults[41], some research suggests that smoking cessation in young people can reduce

cardiometabolic and cardiovascular risk to baseline in as little as five years[42]. This is relevant since PsyMetRiC was developed for younger populations. Therefore, for this reason, and to assist in harmonisation across our development and validation datasets, we included smoking as a binary variable (yes/no). For the SLaM external validation sample, smoking status was derived using the 'CRIS-IE-Smoking' application, which sits within the General Architecture for Text Engineering (GATE) natural language processing software to extract ever smoking status information from open-text fields[43]. For all other samples, smoking was captured as current smoking status from clinical interview.

Prescription of a Metabolically-Active Antipsychotic

Antipsychotic medication is an important contributor to cardiometabolic risk in young people with psychosis, and so it was important to include in PsyMetRiC. Antipsychotic medications have rarely been included in existing cardiometabolic risk prediction algorithms[18]. Three more recent algorithms (QRISK3[44], QDiabetes[45], PRIMROSE[12]) have included antipsychotics as predictors, grouped as binary variables based on the traditional distinctions of typical/atypical or first/second-generation. However, the differential cardiometabolic effects of antipsychotics do not necessarily abide by these distinctions. For example, aripiprazole conveys relatively little adverse cardiometabolic risk, yet olanzapine conveys significant adverse cardiometabolic risk, and both are second generation antipsychotics. Similarly, chlorpromazine conveys significant cardiometabolic risk, yet haloperidol does not, and both are typical antipsychotics. Therefore, we instead grouped antipsychotics based on previous research[24,34] as 'metabolically-active' or not (Supplementary Table 4). This is a notable advance over previous research. Therefore, we classified all individuals who were prescribed a metabolically-active antipsychotic as "1", and all participants who were not prescribed a metabolically-active antipsychotic (including participants who were not prescribed any antipsychotic) as "0". However, we were unable to consider dosage, or the creation of a categorical antipsychotic medication variable including more distinctions of cardiometabolic risk, for several reasons. First, interactions of dosage with antipsychotic choice would have added significant complexity to the model and may have increased the risk of overfit, given our sample size. It would also have been difficult to capture the effect of dosage change on cardiometabolic risk from a single baseline measure of predictor assessment. This is important because antipsychotics are usually commenced at a low dose and upwardly titrated over time depending on treatment response. Second, with increasing numbers of risk-distinguishing categories comes increased subjectivity of group classification for some antipsychotics. In future, when development and validation samples of young people with psychosis are large enough, it would be most appropriate to model the cardiometabolic risk associated with each antipsychotic medication individually.

Blood-based Predictors: HDL and Triglycerides

Blood-based predictors feature less often in cardiometabolic risk prediction algorithms[18]. However, meta-analytic evidence suggests abnormal triglyceride and HDL levels are detectable at the first-episode of psychosis (FEP)[46] even in individuals with limited exposure to antipsychotic medication. A raised triglyceride:HDL ratio is a hall-mark of insulin resistance[47], which is also associated with antipsychotic-naïve FEP[48], whereas meta-analytic evidence suggests that other measures of glucose-insulin homeostasis (e.g. fasting plasma glucose, glycated haemoglobin) are not associated with antipsychotic-naïve FEP[48]. Abnormal HDL[49] and triglycerides[50] are longitudinally associated with cardiometabolic outcomes. Therefore, we chose to include HDL and triglycerides as continuous variables because they are associated with dyslipidaemia in FEP, are associated with long term cardiometabolic outcomes, and are also a useful risk-marker for insulin resistance considering that gold-standard measures for insulin resistance (e.g. homeostatic model assessment for insulin resistance[51]) are rarely carried out in current psychiatric clinical practice.

Supplementary Tables

Supplementary Table 1: Missing Sample Analysis: Model Development Sample (CAMEO)

Variable		Included	Missing	Test Statistic
**Age, mean (SD)**		25.42 (4.77)	28.81 (11.69)	t=5.32, p=0.01
**Sex, *n* (%)**	Male<br>Female	208 (69.57)<br>91 (30.43)	490 (60)<br>324 (40)	χ^2=7.81, p=0.01
**Ethnicity, *n* (%)**	White<br>Black<br>Asian	250 (83.61)<br>15 (5.01)<br>34 (11.37)	676 (83.05)<br>34 (4.18)<br>88 (10.81)	χ^2=0.19, p=0.54
**Smoking, *n* (%)**	Yes<br>No	182 (51.70)<br>117 (48.30)	443 (54.42)<br>371 (45.58)	χ^2=0.15, p=0.70
**Body Mass Index, mean (SD)**		20.53 (8.49)	23.4 (8.80)	t=1.96, p=0.20
**Metabolically Active Antipsychotics, *n* (%)**	Yes<br>No	216 (72.24)<br>83 (27.76)	465 (57.13)<br>349 (42.87)	χ^2=21.04, p=0.01

Missing sample analysis was not conducted for the Birmingham sample since there were no participants that were excluded on the basis of missing data on all exposure/outcome variables; cases were excluded only on the basis of having the outcome at baseline.

Supplementary Table 2: Missing Sample Analysis: External Validation Sample (SLAM)

Variable		Included	Missing	Test Statistic
**Age, mean (SD)**		24.45 (4.75)	29.86 (10.43)	t=18.35, p=0.01
**Sex, *n* (%)**	Male	440 (67.59)	1472 (59.42)	χ^2=15.46, p=0.01
	Female	211 (32.41)	1002 (40.58)	
**Ethnicity, *n* (%)**	White	154 (30.20)	1001 (40.46)	χ^2=18.97, p=0.01
	Black	250 (49.02)	1016 (41.07)	
	Asian	106 (20.78)	458 (18.57)	
**Smoking, *n* (%)**	Yes	469 (91.96)	2029 (81.16)	χ^2=30.81, p=0.01
	No	41 (8.04)	446 (18.84)	
**Body Mass Index, mean (SD)**		22.96 (6.94)	24.38 (6.72)	t=157.41, p=0.01
**Metabolically Active Antipsychotics, *n* (%)**	Yes<br>No	472 (92.55)<br>38 (7.45)	1957 (79.10)<br>518 (21.90)	χ^2=50.68, p=0.01

Supplementary Table 3: Selected Comments From McPin Young Person's Advisory Group (YPAG)

Question Asked To The YPAG	Responses From The YPAG
"Does it surprise you that despite many calculators for diabetes/obesity[a] have been made, none of them have been made for younger people? What do you think about that?"	It is quite worrying because there is strong research evidence that these conditions can develop in young people who have emerging mental health problems. Could be prevented if such a scale was made to lower risk of health issues in later life.
	The calculator could help bring awareness to doctors and young people about the risk.
	Because of the link found with mental health issues which affect all ages, it is important that this calculator is being made.
"On a scale of 1 (not important at all) to 10 (really important), how important do you think it is to know your chance of getting diabetes /obesity[a] in the next 6 years? Why/why not?"	**9** - Because it could help people to make changes to their lifestyle that would prevent them from getting these diseases in the future which would help them to live a longer life. The only reason I didn't put 10 is that some people may not want to know if they are destined to get a disease, even if this is not true, it may not be helpful to some people.
	**5** - It's useful because some people will want to make changes such as exercise more or sleep more to prevent getting these conditions. However, some may find these pointless and counterproductive as the calculator works only by chance.
	**9** – more likely to make those changes if they receive this information
From the information that is asked by the calculator, how happy do you think a young person would be to give that information to a doctor today?	Most people won't have a problem with sharing their height however a lot of people might be uncomfortable sharing their weight because they are unhappy with it
	I don't think that anyone would have a problem sharing this information *[smoking]* unless they are ashamed of how much they smoke
	If there was an option not to have a blood test, it's likely that not many people would opt out
	Weight & sex are quite sensitive subjects

[a]The phrase diabetes/obesity was used in place of metabolic syndrome at YPAG meetings since the former terms are more commonly used in common parlance, and thus more widely understood by non-healthcare professionals.

Supplementary Table 4: Classification of Metabolically-Active Antipsychotics

More Metabolically Active Antipsychotics	Less Metabolically Active Antipsychotics
Olanzapine[34]*	Aripiprazole[34]*
Quetiapine[34]*	Amisulpiride[34]*
Risperidone[34]*	Haloperidol[34]
Paliperidone[34]	Sulpiride[52]
Clozapine[34]	Pericyazine[53]†
Chlorpromazine[34]	Lurasidone[34]†
Asenapine[24]†	Ziprasdone[34]†
Pimozide[52]†	Flupenthixol[24]†
Levomepromazine[52]†	Fluphenazine[24]†
Prochlorperazine[6]†	Zuclopenthixol[52]†
Trifluoperazine[54]†	
Pipotiazine[54]†	

This table comprises all antipsychotics prescribed for participants/patients in all samples.
*indicates the five most commonly prescribed antipsychotics across all samples
†indicates antipsychotics rarely prescribed (<3 participants/patients in total across all samples)

Supplementary Table 5: Decision Curve Analysis Results at Different Thresholds – Full-Model

Net Benefit Performance Measure (95% C.I.)				
**Risk Threshold[a]**	**Sensitivity**	**Specificity**	**Net Benefit**	**Standardized Net Benefit[b]**
0.02	1.00 (1.00-1.00)	0.01 (0.00-0.02)	0.15 (0.13-0.18)	0.90 (0.88-0.92)
0.04	0.99 (0.97-1.00)	0.04 (0.03-0.06)	0.13 (0.11-0.16)	0.80 (0.75-0.83)
0.06	0.99 (0.97-1.00)	0.16 (0.12-0.19)	0.12 (0.09-0.15)	0.73 (0.67-0.77)
0.08	0.96 (0.92-1.00)	0.30 (0.26-0.34)	0.11 (0.09-0.14)	0.66 (0.58-0.72)
0.10	0.94 (0.88-0.98)	0.41 (0.37-0.46)	0.10 (0.08-0.13)	0.62 (0.52-0.69)
0.12	0.92 (0.86-0.97)	0.52 (0.47-0.57)	0.10 (0.07-0.13)	0.60 (0.50-0.68)
0.14	0.85 (0.77-0.91)	0.61 (0.57-0.65)	0.09 (0.06-0.12)	0.53 (0.44-0.62)
0.16	0.76 (0.69-0.83)	0.70 (0.66-0.74)	0.08 (0.06-0.11)	0.48 (0.38-0.59)
0.18	0.75 (0.66-0.82)	0.74 (0.71-0.78)	0.08 (0.05-0.11)	0.47 (0.37-0.58)
0.20	0.68 (0.59-0.77)	0.79 (0.75-0.83)	0.07 (0.05-0.10)	0.42 (0.31-0.53)
0.22	0.62 (0.52-0.70)	0.83 (0.80-0.87)	0.07 (0.04-0.09)	0.39 (0.27-0.49)
0.24	0.56 (0.47-0.65)	0.86 (0.83-0.89)	0.06 (0.04-0.08)	0.35 (0.22-0.49)
0.26	0.52 (0.43-0.62)	0.88 (0.85-0.91)	0.05 (0.03-0.07)	0.31 (0.19-0.43)
0.28	0.45 (0.37-0.54)	0.90 (0.87-0.92)	0.04 (0.02-0.07)	0.26 (0.15-0.38)
0.30	0.40 (0.31-0.50)	0.92 (0.89-0.94)	0.04 (0.02-0.06)	0.23 (0.12-0.36)
0.32	0.37 (0.28-0.47)	0.93 (0.90-0.95)	0.03 (0.02-0.06)	0.20 (0.10-0.32)
0.34	0.34 (0.24-0.43)	0.94 (0.92-0.96)	0.03 (0.01-0.05)	0.19 (0.08-0.30)
0.36	0.27 (0.19-0.36)	0.95 (0.94-0.97)	0.02 (0.01-0.04)	0.14 (0.04-0.26)

[a]Different risk thresholds may be selected depending on the proposed intervention (i.e., balancing the risk/benefit of exposing false positives to an intervention to benefit the most true positives), as well as patient or clinician preference.
[b]Standardized net benefit is calculated as the net benefit / outcome prevalence, showing the proportion of improvement in net benefit at the selected risk threshold.

Supplementary Table 6: Decision Curve Analysis Results at Different Thresholds – Partial-Model

Net Benefit Performance Measure (95% C.I.)				
**Risk Threshold[a]**	**Sensitivity**	**Specificity**	**Net Benefit**	**Standardized Net Benefit[b]**
0.02	1.00 (1.00-1.00)	0.01 (0.00-0.01)	0.15 (0.12-0.18)	0.90 (0.88-0.92)
0.04	1.00 (1.00-1.00)	0.03 (0.02-0.05)	0.14 (0.11-0.16)	0.80 (0.75-0.83)
0.06	0.99 (0.96-1.00)	0.13 (0.10-0.15)	0.12 (0.09-0.15)	0.72 (0.64-0.77)
0.08	0.99 (0.96-1.00)	0.24 (0.21-0.28)	0.11 (0.08-0.14)	0.67 (0.58-0.73)
0.10	0.95 (0.91-0.99)	0.38 (0.34-0.43)	0.10 (0.07-0.13)	0.62 (0.53-0.69)
0.12	0.91 (0.86-0.96)	0.50 (0.46-0.54)	0.10 (0.07-0.12)	0.57 (0.47-0.65)
0.14	0.85 (0.78-0.91)	0.58 (0.53-0.62)	0.09 (0.06-0.11)	0.51 (0.38-0.59)
0.16	0.78 (0.71-0.86)	0.66 (0.62-0.70)	0.08 (0.05-0.11)	0.46 (0.33-0.55)
0.18	0.75 (0.65-0.83)	0.74 (0.70-0.77)	0.08 (0.05-0.10)	0.46 (0.33-0.56)
0.20	0.67 (0.60-0.75)	0.79 (0.76-0.83)	0.07 (0.04-0.09)	0.42 (0.30-0.51)
0.22	0.65 (0.56-0.72)	0.82 (0.79-0.86)	0.07 (0.04-0.09)	0.40 (0.27-0.50)
0.24	0.59 (0.50-0.67)	0.86 (0.83-0.90)	0.06 (0.04-0.08)	0.37 (0.25-0.48)
0.26	0.56 (0.47-0.65)	0.87 (0.85-0.91)	0.06 (0.03-0.08)	0.34 (0.23-0.44)
0.28	0.48 (0.40-0.57)	0.89 (0.86-0.92)	0.04 (0.02-0.07)	0.26 (0.13-0.37)
0.30	0.41 (0.34-0.50)	0.91 (0.89-0.94)	0.04 (0.02-0.06)	0.23 (0.11-0.33)
0.32	0.35 (0.28-0.44)	0.92 (0.90-0.94)	0.03 (0.01-0.05)	0.17 (0.06-0.27)
0.34	0.29 (0.21-0.38)	0.94 (0.92-0.96)	0.02 (0.00-0.04)	0.13 (0.02-0.24)
0.36	0.28 (0.20-0.36)	0.94 (0.92-0.96)	0.02 (0.00-0.04)	0.12 (0.01-0.22)

[a]Different risk thresholds may be preferred depending on the proposed intervention (i.e., balancing the risk/benefit of the intervention), as well as patient or clinician preference.

[b]Standardized net benefit is calculated as the net benefit / outcome prevalence, showing the %improvement in net benefit at the selected risk threshold.

Supplementary Figures

Supplementary Figure 1: Histograms of Predicted Outcome Probabilities in Algorithm Development Sample after Coefficient Shrinkage

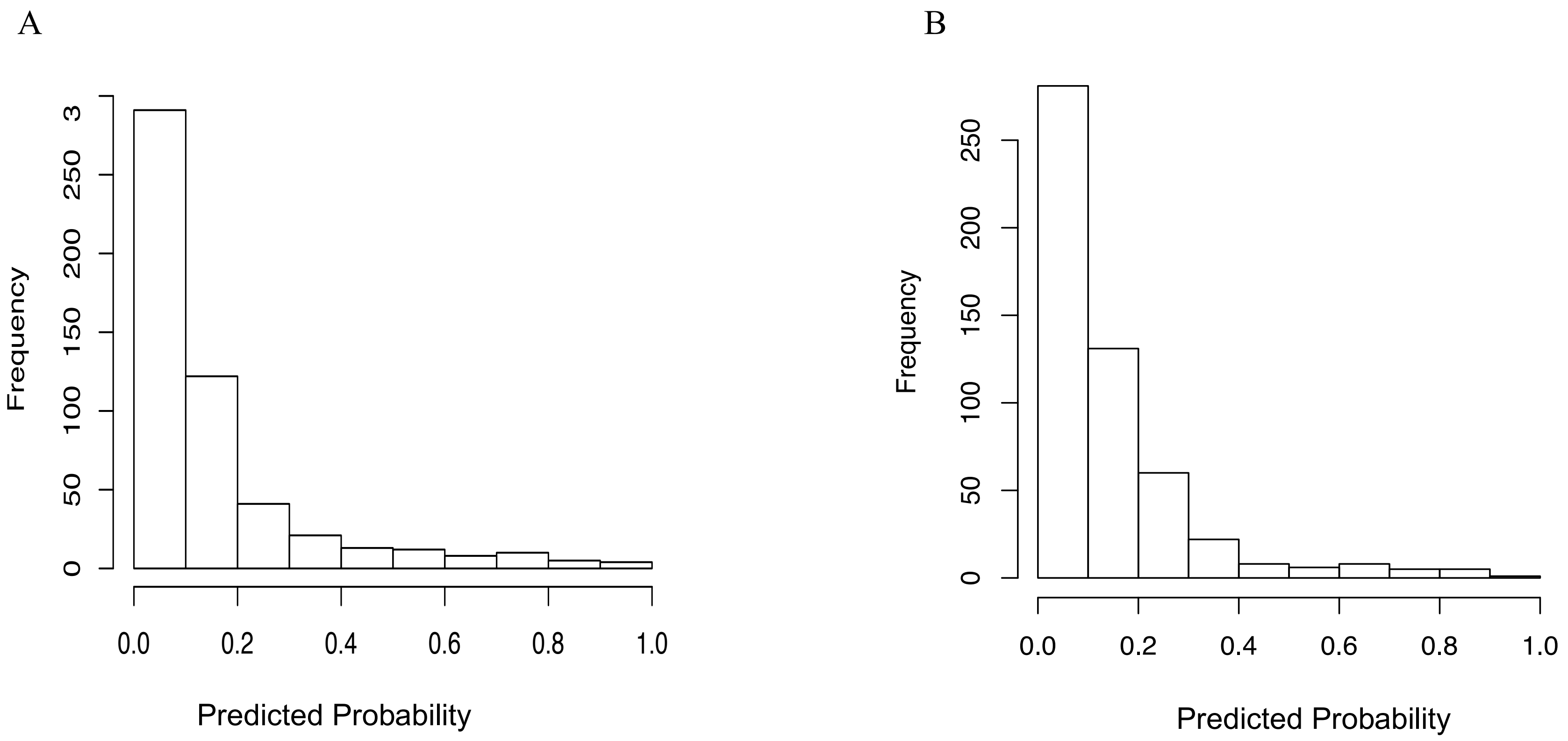

A=Full-Model; B=Partial-Model

Supplementary Figure 2: Internal Validation Calibration Plots in Development Sample

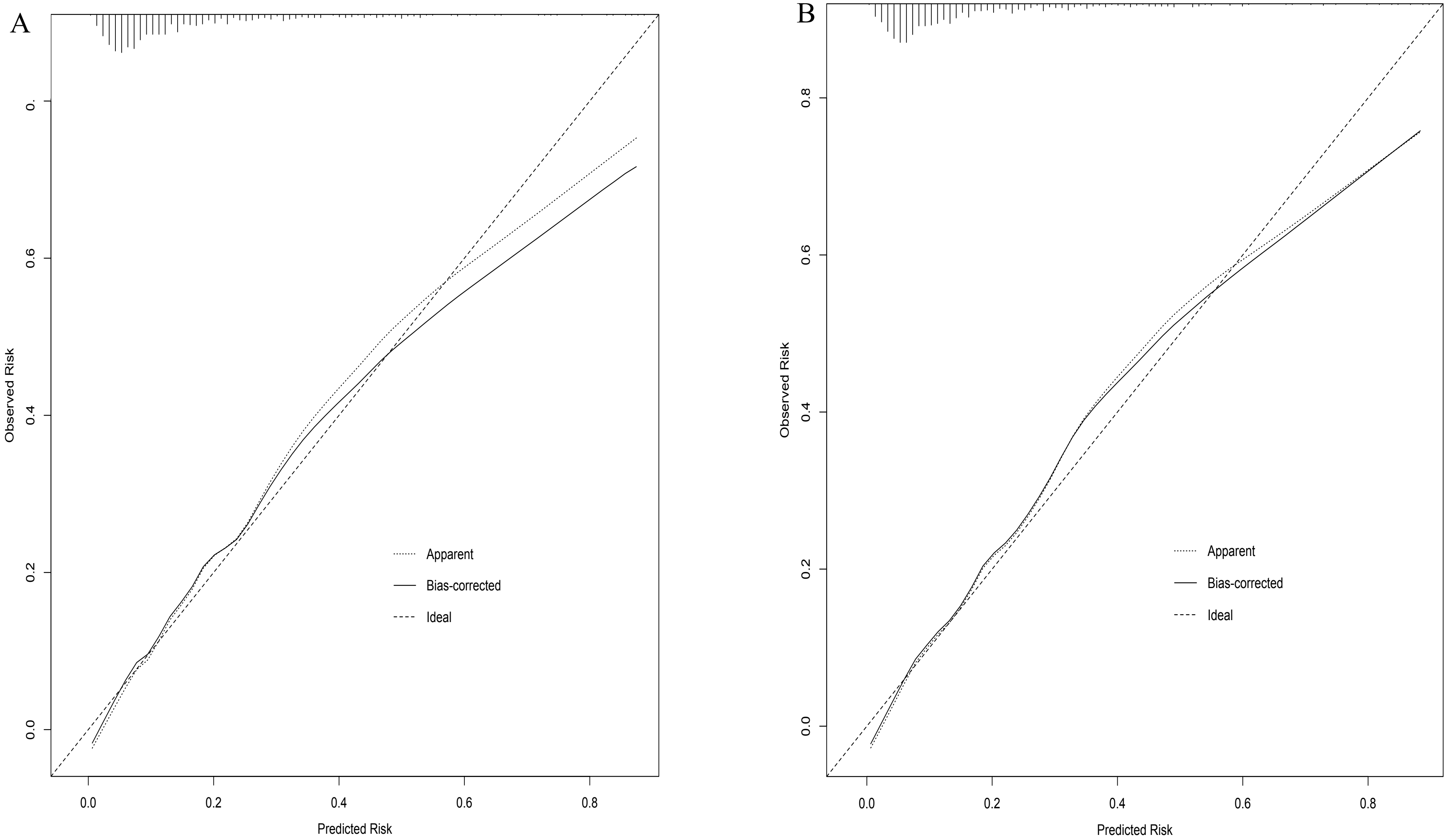

A=Full-Model; B=Partial-Model

Calibration plots illustrate agreement between observed risk (y axis) and expected risk (x axis). Perfect agreement would trace the dotted “ideal” line. Algorithm calibration is illustrated by the dotted (Apparent) and solid (Bias Corrected) lines.

Supplementary Figure 3: Histograms of Predicted Outcome Probabilities in External Validation Sample

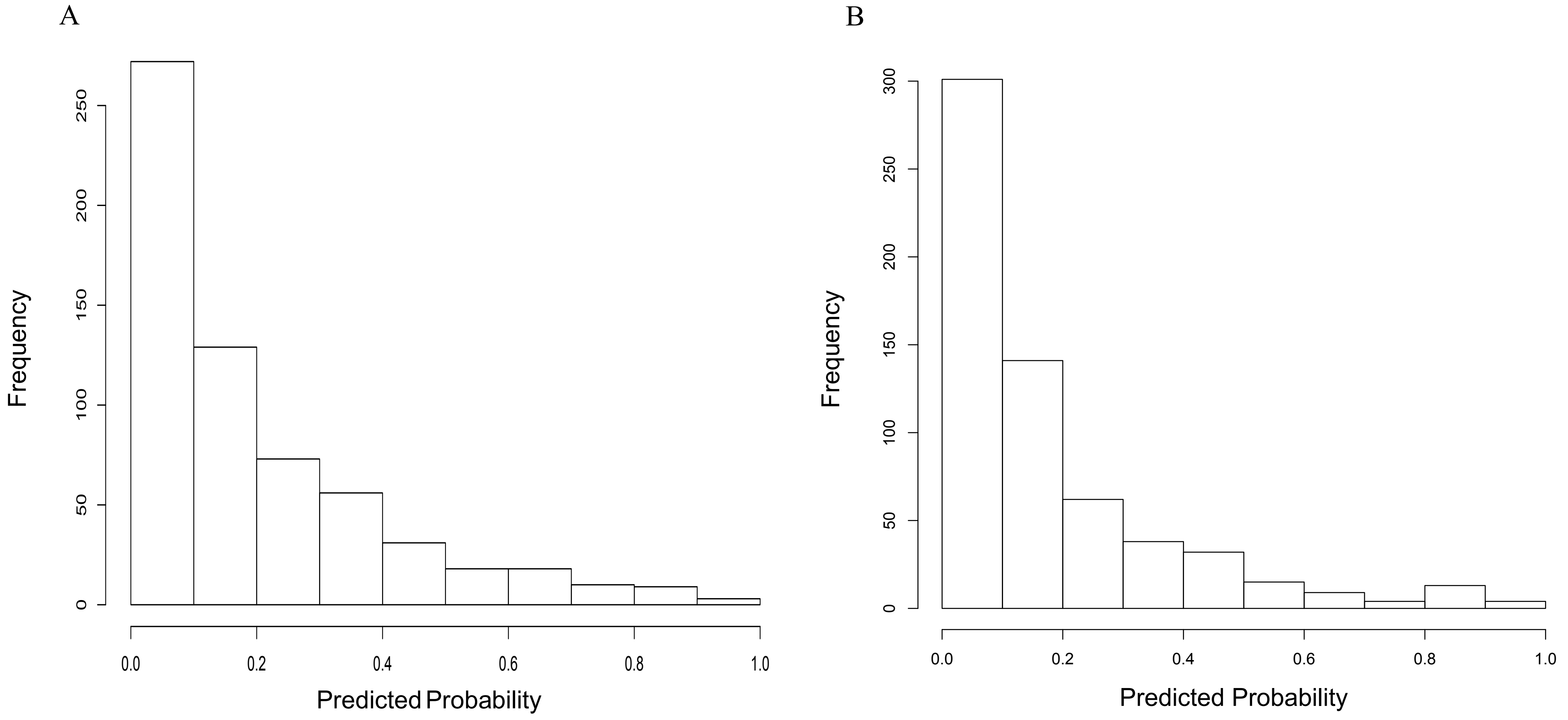

A=Full-Model; B=Partial-Model

Supplementary Figure 4: Histograms of Predicted Outcome Probabilities in ALSPAC Sensitivity Analysis Sample

A

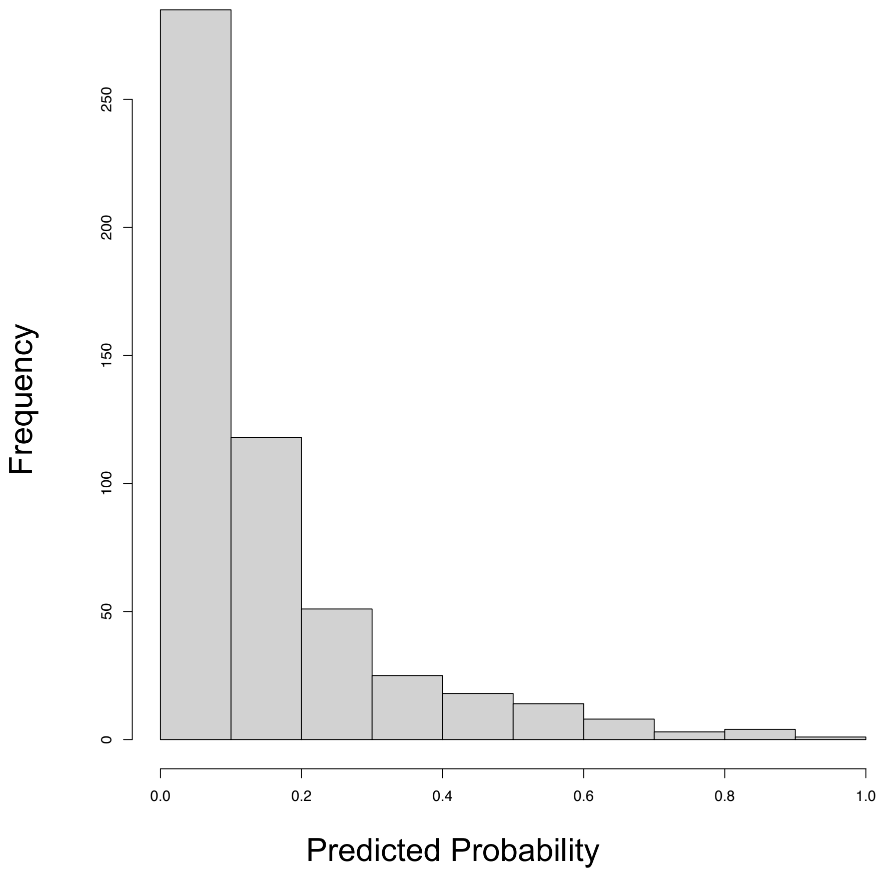

B

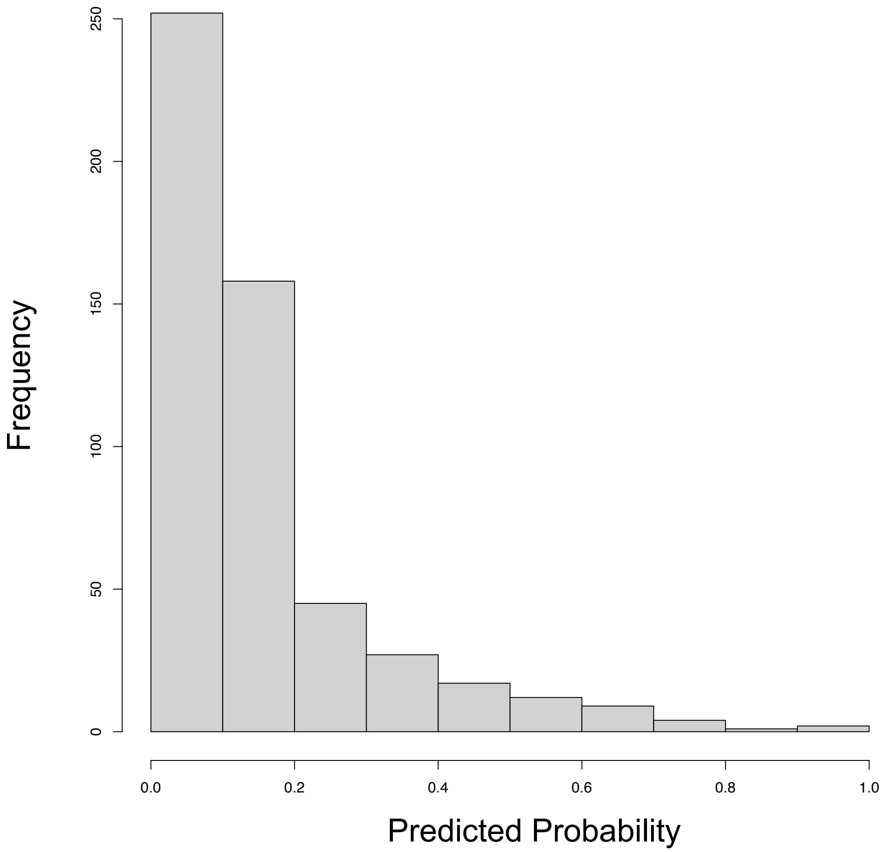

Supplementary Figure 5: Calibration Plots in ALSPAC Sensitivity Analysis Sample

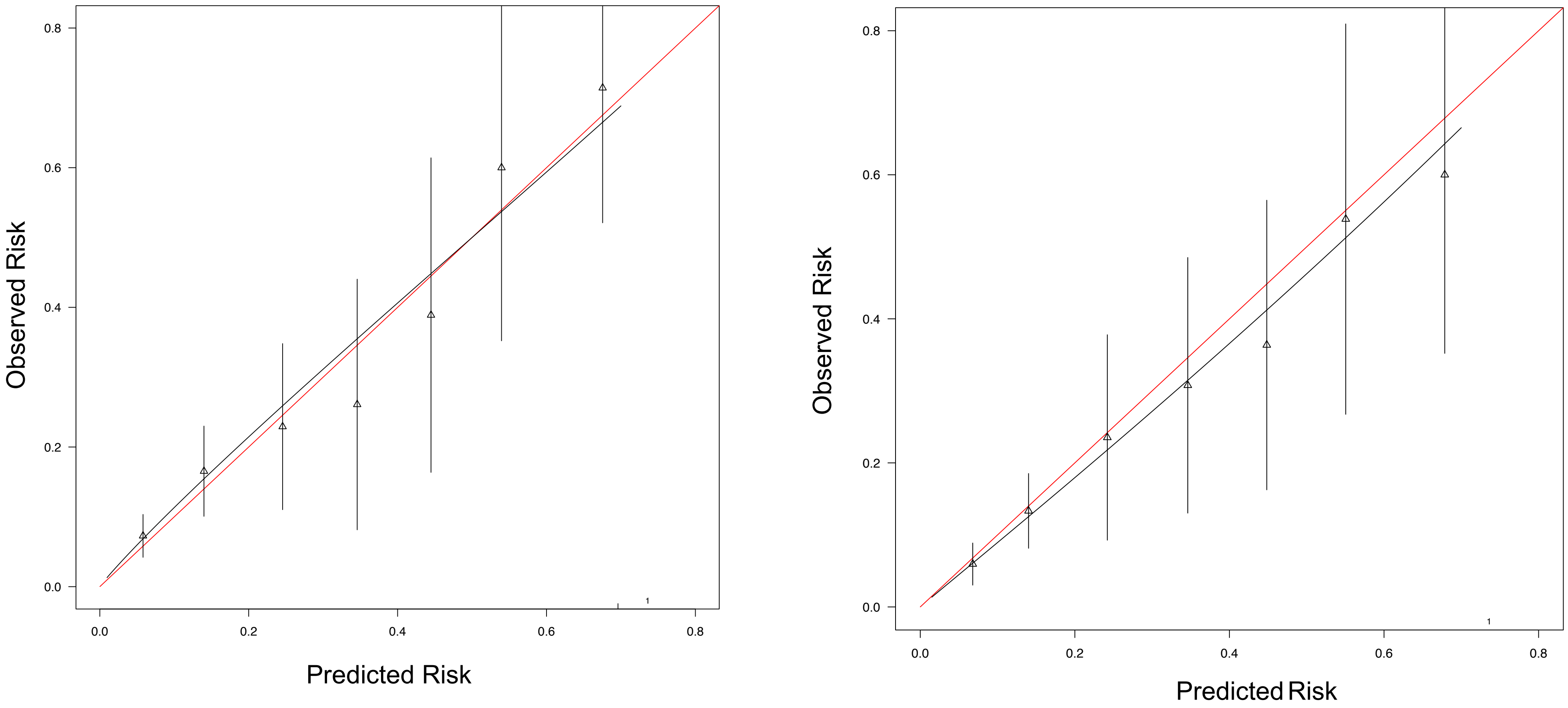

A=Full-Model; B=Partial-Model.
Calibration plots illustrate agreement between observed risk (y axis) and predicted risk (x axis). Perfect agreement would trace the red line. Algorithm calibration is illustrated by the black line. Triangles denote grouped observations for participants at deciles of predicted risk, with 95% C.I.'s indicated by the vertical black lines.

Supplementary Data – TRIPOD Checklist: Prediction Model Development & Validation

Section/Topic			Checklist Item	Section/ Paragraph
**Title and abstract**				
Title	1	D;V	Identify the study as developing and/or validating a multivariable prediction model, the target population, and the outcome to be predicted.	Title
Abstract	2	D;V	Provide a summary of objectives, study design, setting, participants, sample size, predictors, outcome, statistical analysis, results, and conclusions.	Abstract
**Introduction**				
Background and objectives	3a	D;V	Explain the medical context (including whether diagnostic or prognostic) and rationale for developing or validating the multivariable prediction model, including references to existing models.	Introduction Paragraphs 1-2
	3b	D;V	Specify the objectives, including whether the study describes the development or validation of the model or both.	Introduction Paragraph 3
**Methods**				
Source of data	4a	D;V	Describe the study design or source of data (e.g., randomized trial, cohort, or registry data), separately for the development and validation data sets, if applicable.	Methods – Data Sources – Paragraph 1-3
	4b	D;V	Specify the key study dates, including start of accrual; end of accrual; and, if applicable, end of follow-up.	Methods – Data Sources – Paragraph 1-3
Participants	5a	D;V	Specify key elements of the study setting (e.g., primary care, secondary care, general population) including number and location of centres.	Methods – Data Sources – Paragraph 1-3
	5b	D;V	Describe eligibility criteria for participants.	Methods – Data Sources – Paragraph 1-3
	5c	D;V	Give details of treatments received, if relevant.	N/A
Outcome	6a	D;V	Clearly define the outcome that is predicted by the prediction model, including how and when assessed.	Methods – Outcome – Paragraph 1
	6b	D;V	Report any actions to blind assessment of the outcome to be predicted.	N/A (retrospective analysis)
Predictors	7a	D;V	Clearly define all predictors used in developing or validating the multivariable prediction model, including how and when they were measured.	Methods – Data Sources – Paragraph 1-3; Methods – Predictor Variables – Paragraph 1
	7b	D;V	Report any actions to blind assessment of predictors for the outcome and other predictors.	N/A (retrospective analysis)
Sample size	8	D;V	Explain how the study size was arrived at.	Methods – Data Sources – Paragraph 1-3
Missing data	9	D;V	Describe how missing data were handled (e.g., complete-case analysis, single imputation, multiple imputation) with details of any imputation method.	Methods – Statistical Analysis – Paragraph 1
Statistical analysis methods	10a	D	Describe how predictors were handled in the analyses.	Methods – Statistical Analysis – Paragraph 1
	10b	D	Specify type of model, all model-building procedures (including any predictor selection), and method for internal validation.	Methods – Statistical Analysis – Paragraph 1
	10c	V	For validation, describe how the predictions were calculated.	Methods – Statistical Analysis – Paragraph 2
	10d	D;V	Specify all measures used to assess model performance and, if relevant, to compare multiple models.	Methods – Statistical

				Analysis – Paragraph 2
	10e	V	Describe any model updating (e.g., recalibration) arising from the validation, if done.	N/A
Risk groups	11	D;V	Provide details on how risk groups were created, if done.	N/A
Development vs. validation	12	V	For validation, identify any differences from the development data in setting, eligibility criteria, outcome, and predictors.	Table 1
**Results**				
Participants	13a	D;V	Describe the flow of participants through the study, including the number of participants with and without the outcome and, if applicable, a summary of the follow-up time.	Methods – Data Sources – Paragraph 1-3; Table 1
	13b	D;V	Describe the characteristics of the participants (basic demographics, clinical features, available predictors).	Table 1
	13c	V	For validation, show a comparison with the development data of the distribution of important variables (demographics, predictors and outcome).	Table 1
Model development	14a	D	Specify the number of participants and outcome events in each analysis.	Table 1
	14b	D	If done, report the unadjusted association between each candidate predictor and outcome.	N/A
Model specification	15a	D	Present the full prediction model to allow predictions for individuals (i.e., all regression coefficients, and model intercept or baseline survival at a given time point).	Table 2
	15b	D	Explain how to the use the prediction model.	Methods – Statistical Analysis – Paragraph 1; Online Data Visualisation App
Model performance	16	D;V	Report performance measures (with CIs) for the prediction model.	Results – Paragraphs 2-5
Model-updating	17	V	If done, report the results from any model updating (i.e., model specification, model performance).	N/A
**Discussion**				
Limitations	18	D;V	Discuss any limitations of the study (such as nonrepresentative sample, few events per predictor, missing data).	Discussion – Paragraph 11
Interpretation	19a	V	For validation, discuss the results with reference to performance in the development data, and any other validation data.	Discussion – Paragraph 1
	19b	D;V	Give an overall interpretation of the results, considering objectives, limitations, results from similar studies, and other relevant evidence.	Discussion – Paragraphs 1-11
Implications	20	D;V	Discuss the potential clinical use of the model and implications for future research.	Discussion – Paragraphs 1-11
**Other information**				
Supplementary information	21	D;V	Provide information about the availability of supplementary resources, such as study protocol, Web calculator, and data sets.	Results – Paragraph 6; Data Availability Statement
Funding	22	D;V	Give the source of funding and the role of the funders for the present study.	Funding Statement

References

1. Boyd A, Golding J, Macleod J, et al. Cohort Profile: The 'Children of the 90s'--the index offspring of the Avon Longitudinal Study of Parents and Children. *International journal of epidemiology* 2013; **42**(1): 111-27.
2. Fraser A, Macdonald-Wallis C, Tilling K, et al. Cohort Profile: the Avon Longitudinal Study of Parents and Children: ALSPAC mothers cohort. *Int J Epidemiol* 2013; **42**(1): 97-110.
3. Northstone K, Lewcock M, Groom A, et al. The Avon Longitudinal Study of Parents and Children (ALSPAC): an update on the enrolled sample of index children in 2019. *Wellcome Open Res* 2019; **4**: 51.
4. Harris PA, Taylor R, Minor BL, et al. The REDCap consortium: Building an international community of software platform partners. *J Biomed Inform* 2019; **95**: 103208.
5. Harris PA, Taylor R, Thielke R, Payne J, Gonzalez N, Conde JG. Research electronic data capture (REDCap)--a metadata-driven methodology and workflow process for providing translational research informatics support. *J Biomed Inform* 2009; **42**(2): 377-81.
6. Sullivan SA, Kounali D, Cannon M, et al. A Population-Based Cohort Study Examining the Incidence and Impact of Psychotic Experiences From Childhood to Adulthood, and Prediction of Psychotic Disorder. *Am J Psychiatry* 2020: appiajp201919060654.
7. Zammit S, Kounali D, Cannon M, et al. Psychotic Experiences and Psychotic Disorders at Age 18 in Relation to Psychotic Experiences at Age 12 in a Longitudinal Population-Based Cohort Study. *The American journal of psychiatry* 2013; **170**(7): 742-50.
8. Riley RD, Snell KI, Ensor J, et al. Minimum sample size for developing a multivariable prediction model: PART II - binary and time-to-event outcomes. *Stat Med* 2019; **38**(7): 1276-96.
9. Ensor J, Martin, E.C., Riley, R.D.,. pmsampsize: Calculates the Minimum Sample Size Required for Developing a Multivariable Prediction Model. https://CRAN.R-project.org/package=pmsampsize; 2019.
10. R Core Team. R: A Language and Environment for Statistical Computing.2017. https://www.R-project.org/ (accessed.
11. Mitchell AJ, Vancampfort D, Sweers K, van Winkel R, Yu W, De Hert M. Prevalence of metabolic syndrome and metabolic abnormalities in schizophrenia and related disorders--a systematic review and meta-analysis. *Schizophr Bull* 2013; **39**(2): 306-18.
12. Osborn DP, Hardoon S, Omar RZ, et al. Cardiovascular risk prediction models for people with severe mental illness: results from the prediction and management of cardiovascular risk in people with severe mental illnesses (PRIMROSE) research program. *JAMA Psychiatry* 2015; **72**(2): 143-51.
13. Buuren Sv, Groothuis-Oudshoorn, K. MICE: Multivariate Imputation by Chained Equations in R. *Journal of Statistical Software* 2011; **45**(3): 1-67.
14. Lee HJ, Huber, J.,. Multiple imputation with large proportions of missing data: How much is too much? United Kingdom Stata Users' Group Meetings 2011; 2011: Stata Users Group; 2011.
15. Dong Y, Peng CY. Principled missing data methods for researchers. *Springerplus* 2013; **2**(1): 222.
16. Madley-Dowd P, Hughes R, Tilling K, Heron J. The proportion of missing data should not be used to guide decisions on multiple imputation. *J Clin Epidemiol* 2019; **110**: 63-73.
17. Fukuma S, Shimizu S, Shintani A, Kamitani T, Akizawa T, Fukuhara S. Development and validation of a prediction model for loss of physical function in elderly hemodialysis patients. *Nephrol Dial Transplant* 2018; **33**(8): 1452-8.
18. Perry BI, Upthegrove R, Crawford O, et al. Cardiometabolic Risk Prediction Algorithms for Young People with Psychosis: A Systematic Review and Exploratory Analysis. *Acta Psychiatr Scand* 2020.
19. Subramanian J, Simon R. Overfitting in prediction models - is it a problem only in high dimensions? *Contemp Clin Trials* 2013; **36**(2): 636-41.
20. Harrell FE, Jr. Regressing Model Strategies: With Applications to Linearc Models, Logistic Regression, and Survival Analysis. New York: Springer-Verlag; 2001.

21. Steyerberg EW, Eijkemans MJ, Harrell FE, Jr., Habbema JD. Prognostic modelling with logistic regression analysis: a comparison of selection and estimation methods in small data sets. *Stat Med* 2000; **19**(8): 1059-79.
22. Lever J, Krzywinski, M., Altman, N. Model Selection and Overfitting. *Nature Methods* 2016; **13**: 703-4.
23. Deboer MD. Ethnicity, obesity and the metabolic syndrome: implications on assessing risk and targeting intervention. *Expert Rev Endocrinol Metab* 2011; **6**(2): 279-89.
24. Pillinger T, McCutcheon RA, Vano L, et al. Comparative effects of 18 antipsychotics on metabolic function in patients with schizophrenia, predictors of metabolic dysregulation, and association with psychopathology: a systematic review and network meta-analysis. *Lancet Psychiatry* 2020; **7**(1): 64-77.
25. Tillin T, Forouhi N, Johnston DG, McKeigue PM, Chaturvedi N, Godsland IF. Metabolic syndrome and coronary heart disease in South Asians, African-Caribbeans and white Europeans: a UK population-based cross-sectional study. *Diabetologia* 2005; **48**(4): 649-56.
26. Nestel P, Lyu R, Low LP, et al. Metabolic syndrome: recent prevalence in East and Southeast Asian populations. *Asia Pac J Clin Nutr* 2007; **16**(2): 362-7.
27. Lear SA, Gasevic D. Ethnicity and Metabolic Syndrome: Implications for Assessment, Management and Prevention. *Nutrients* 2019; **12**(1).
28. Pradhan AD. Sex differences in the metabolic syndrome: implications for cardiovascular health in women. *Clin Chem* 2014; **60**(1): 44-52.
29. Kuk JL, Ardern CI. Age and sex differences in the clustering of metabolic syndrome factors: association with mortality risk. *Diabetes Care* 2010; **33**(11): 2457-61.
30. Kim SH, Reaven G. Sex differences in insulin resistance and cardiovascular disease risk. *J Clin Endocrinol Metab* 2013; **98**(11): E1716-21.
31. Gupte AA, Pownall HJ, Hamilton DJ. Estrogen: an emerging regulator of insulin action and mitochondrial function. *J Diabetes Res* 2015; **2015**: 916585.
32. Beale AL, Meyer P, Marwick TH, Lam CSP, Kaye DM. Sex Differences in Cardiovascular Pathophysiology: Why Women Are Overrepresented in Heart Failure With Preserved Ejection Fraction. *Circulation* 2018; **138**(2): 198-205.
33. Van Gaal LF, Mertens IL, De Block CE. Mechanisms linking obesity with cardiovascular disease. *Nature* 2006; **444**(7121): 875-80.
34. Leucht S, Cipriani A, Spineli L, et al. Comparative efficacy and tolerability of 15 antipsychotic drugs in schizophrenia: a multiple-treatments meta-analysis. *Lancet* 2013; **382**(9896): 951-62.
35. Spertus J, Horvitz-Lennon M, Abing H, Normand SL. Risk of weight gain for specific antipsychotic drugs: a meta-analysis. *NPJ Schizophr* 2018; **4**(1): 12.
36. Shah NR, Braverman ER. Measuring adiposity in patients: the utility of body mass index (BMI), percent body fat, and leptin. *PLoS One* 2012; **7**(4): e33308.
37. Barreira TV, Harrington DM, Staiano AE, Heymsfield SB, Katzmarzyk PT. Body adiposity index, body mass index, and body fat in white and black adults. *JAMA* 2011; **306**(8): 828-30.
38. Banks E, Joshy G, Korda RJ, et al. Tobacco smoking and risk of 36 cardiovascular disease subtypes: fatal and non-fatal outcomes in a large prospective Australian study. *BMC Med* 2019; **17**(1): 128.
39. Polubriaginof F, Salmasian H, Albert DA, Vawdrey DK. Challenges with Collecting Smoking Status in Electronic Health Records. *AMIA Annu Symp Proc* 2017; **2017**: 1392-400.
40. Duncan MS, Freiberg MS, Greevy RA, Jr., Kundu S, Vasan RS, Tindle HA. Association of Smoking Cessation With Subsequent Risk of Cardiovascular Disease. *JAMA* 2019; **322**(7): 642-50.
41. Mons U, Muezzinler A, Gellert C, et al. Impact of smoking and smoking cessation on cardiovascular events and mortality among older adults: meta-analysis of individual participant data from prospective cohort studies of the CHANCES consortium. *BMJ* 2015; **350**: h1551.
42. Lloyd-Jones DM, Huffman MD, Karmali KN, et al. Estimating Longitudinal Risks and Benefits From Cardiovascular Preventive Therapies Among Medicare Patients: The Million Hearts

Longitudinal ASCVD Risk Assessment Tool: A Special Report From the American Heart Association and American College of Cardiology. *Circulation* 2017; **135**(13): e793-e813.
43. Wu CY, Chang CK, Robson D, et al. Evaluation of smoking status identification using electronic health records and open-text information in a large mental health case register. *PLoS One* 2013; **8**(9): e74262.
44. Hippisley-Cox J, Coupland C, Brindle P. Development and validation of QRISK3 risk prediction algorithms to estimate future risk of cardiovascular disease: prospective cohort study. *BMJ* 2017; **357**: j2099.
45. Hippisley-Cox J, Coupland C. Development and validation of QDiabetes-2018 risk prediction algorithm to estimate future risk of type 2 diabetes: cohort study. *BMJ* 2017; **359**: j5019.
46. Misiak B, Stanczykiewicz B, Laczmanski L, Frydecka D. Lipid profile disturbances in antipsychotic-naive patients with first-episode non-affective psychosis: A systematic review and meta-analysis. *Schizophr Res* 2017.
47. Murguia-Romero M, Jimenez-Flores JR, Sigrist-Flores SC, et al. Plasma triglyceride/HDL-cholesterol ratio, insulin resistance, and cardiometabolic risk in young adults. *J Lipid Res* 2013; **54**(10): 2795-9.
48. Perry BI, McIntosh G, Weich S, Singh S, Rees K. The association between first-episode psychosis and abnormal glycaemic control: systematic review and meta-analysis. *Lancet Psychiatry* 2016; **3**(11): 1049-58.
49. Rader DJ, Hovingh GK. HDL and cardiovascular disease. *Lancet* 2014; **384**(9943): 618-25.
50. Nordestgaard BG, Varbo A. Triglycerides and cardiovascular disease. *Lancet* 2014; **384**(9943): 626-35.
51. Levy JC, Matthews DR, Hermans MP. Correct homeostasis model assessment (HOMA) evaluation uses the computer program. *Diabetes Care* 1998; **21**(12): 2191-2.
52. Bak M, Fransen A, Janssen J, van Os J, Drukker M. Almost all antipsychotics result in weight gain: a meta-analysis. *PLoS One* 2014; **9**(4): e94112.
53. Matar HE, Almerie MQ, Makhoul S, Xia J, Humphreys P. Pericyazine for schizophrenia. *Cochrane Database Syst Rev* 2014; (5): CD007479.
54. Alonso-Pedrero L, Bes-Rastrollo M, Marti A. Effects of antidepressant and antipsychotic use on weight gain: A systematic review. *Obes Rev* 2019; **20**(12): 1680-90.
